# Analysis of genetic diversity of *Zymoseptoria tritici* populations in central and south-eastern Ethiopia

**DOI:** 10.3389/fpls.2025.1505455

**Published:** 2025-04-09

**Authors:** Ayantu Tucho, Tilahun Mekonnen, Farideh Ghadamgahi, Samrat Ghosh, Diriba Muleta, Kassahun Tesfaye, Eu Shang Wang, Tesfaye Alemu, Ramesh Raju Vetukuri

**Affiliations:** ^1^ Biotechnology Research Centre, Institute of Advanced Science and Technology (IAST), Addis Ababa University, Addis Ababa, Ethiopia; ^2^ Department of Plant Science, Salale University, Fitche, Ethiopia; ^3^ Department of Plant Breeding, Swedish University of Agricultural Sciences, Lomma, Sweden; ^4^ Department of Microbial, Cellular and Molecular Biology, Addis Ababa University, Ethiopia University, Addis Ababa, Ethiopia; ^5^ Department of Microbial Sciences and Genetics, College of Natural and Computational Sciences, Bio and Emerging Technology Institute (BETin), Addis Ababa University, Addis Ababa, Ethiopia

**Keywords:** AMOVA, gene flow, genetic analysis, ITS rDNA, SSR, structure, *Z. tritici*

## Abstract

Septoria tritici blotch (STB), caused by the hemibiotrophic fungus *Zymoseptoria tritici*, is a serious threat to global wheat production, and a major bottleneck to wheat production in Ethiopia. Accurate identification and analysis of the pathogen’s genetic structure helps to develop robust STB management strategies. This study aimed at molecular identification and genetic structure analysis of 200 isolates of *Z. tritici* representing six populations in central and south-eastern regions of Ethiopia. A total of 165 isolates were confirmed by Sanger sequencing of the internal transcribed spacer (ITS) region of nuclear DNA (rDNA) region. The pathogen’s genetic structure was further examined using 12 simple sequence repeat (SSR) markers. The microsatellite markers were highly polymorphic and informative, with mean number of alleles (Na), effective alleles (Ne), Nei’s gene diversity of 6.23, 2.90, and 0.59, respectively. Analysis of molecular variance (AMOVA) confirmed the presence of low population differentiation (F_ST_ = 0.02), high gene flow (Nm = 14.7), with 95% of the total genetic variation residing within populations, and leaving only 5% for the among populations. The highest genetic diversity (Number of allele = 9.33, Effective number of allele = 3.4 and Nei’s gene diversity = 0.68) was observed in the Oromia special zone surrounding Finfinnee (OSZ) *Z. tritici* populations, followed by Arsi and North Shewa populations, indicating that these areas are ideal for multi-location wheat germplasm resistance screening, and also the pathogen genetic and genomic analyses. Cluster analyses did not clearly divide the populations into genetically separate clusters according to their geographic areas of sampling, probably due to high gene flow. The analysis revealed existence of high genetic admixture, and all the individuals shared genomic backgrounds from two subgroups (K=2). Overall, the SSR markers are highly informative and effective genetic tools for unlocking the pathogen’s genetic structure. The *Z. tritici* populations of central and southeast Ethiopia exhibit high genetic diversity, indicating the need to deploy durable and diverse disease management strategies. North Shewa, OSZ, Arsi and West Arsi administrative zones represent hotspots for genetic and genomic analyses of *Z. tritici* and excellent locations for host–pathogen interaction studies, and wheat germplasm screening for STB resistance.

## 1 Introduction

Wheat (*Triticum aestivum* L.) is one of the most important global cereals, supporting nearly 35% of the world’s population as a staple food (statista, 2023). South Africa and Ethiopia are the largest wheat producers in Sub-Saharan Africa (Dibaba, 2019). In Ethiopia, wheat ranks fourth after teff (*Eragrostis tef*), maize (*Zea mays*) and sorghum (*Sorghum bicolor*) in area coverage, and third after maize and teff in total production (Bezabih et al., 2023). It is cultivated by nearly five million householders, on about 1.7 million ha, for various purposes, including as food, animal feed and income generation (Latta et al., 2013). Bread wheat accounts for nearly 80% of the total wheat production of the country (Bezabeh et al., 2014; Latta et al., 2013). In 2022, 2.3 million ha of Ethiopia was covered with wheat, with a total production and productivity of seven million metric tonne and 3.04 t/ha, respectively (FAOSTAT, 2024). This national average productivity (3.04 t/ha) is far lower than the global average of 3.69 t/ha (FAOSTAT, 2024) and the productivity level in USA (3.5 t/ha), resulting in a production limit to meet the growing demand for food by the ever-increasing population of the country (Gemechu and Tadese, 2018). Limited access to advanced production technologies, low agricultural inputs (fungicides, improved varieties and fertilizer), and biotic and abiotic stresses are some of the major wheat production constraints in Ethiopia.

In Ethiopia, wheat cultivation is persistently affected by over 30 fungal diseases (Badebo et al., 2006), of which stripe (yellow) rust caused by *Puccinia striiformis* f. sp. *tritici* (Pst), stem rust caused by *P. graminis* f. sp. *tritici*), leaf rust caused by *P. triticina*, and STB caused by *Z. tritici* are the most destructive diseases (Hailu et al., 2015; Huluka, 2002; Mann and Warner, 2017). Rusts can result in grain yield losses of 60–100% (Olivera et al., 2015). STB can cause 25 – 82% wheat production loss in the worst seasons, and the disease incidence and severity has increased recently in Ethiopia’s major wheat belts (Mekonnen et al., 2020). STB infestation results in significant grain yield loss by reducing tillering, poor seed set, poor grain fill or shriveled kernels, and death of leaves, spikes or the entire plant (Goodwin et al., 2011; Mekonnen et al., 2020).

*Zymoseptoria tritici* (*Mycosphaerella graminicola*), the causal agent of septoria tritici blotch (STB) of wheat (McDonald et al., 2015), is a hemibiotrophic apoplastic fungal species (Ma et al., 2018). It belongs to the class Ascomycete (Testa et al., 2015), and has a haploid genome of 21 chromosomes: 13 core chromosomes (which are 1.2−6.1 Mb in size and conserved across *Z. tritici* isolates) and 8 accessory chromosomes which vary in number and size (0.4−0.8 Mb) among *Z. tritici* isolates (Goodwin et al., 2011). The pathogen’s genome varies from 37.1 to 41.7 Mb depending on geographic origins (Goodwin et al., 2011). *Z. tritici* possesses a heterothallic mating system, which requires two compatible partners of opposing mating types (mat1-1 and mat1-2) to come together to create sexual spores (Waalwijk et al., 2002). STB is the most destructive disease of wheat across the world, causing yield losses of up to 50% in untreated fields planted with susceptible cultivars (Eyal and Levy, 1987; McDonald et al., 2015). Primary infections of STB can occur from airborne or rain-splashed asexual pycnidospores and sexual ascospores from infected crop debris (Hunter et al., 1999).

The narrow genetic basis of modern wheat cultivars and rapidly changing fungal genomes may have resulted in the frequent breakdown of host resistance (Ye et al., 2019). The sexual cycle of *Z. tritici*, synonyms *M. graminicola* plays a crucial role in its epidemiology and genetic diversity among field isolates. Resistance breeding provides the best and eco-friendly approach to managing STB. Understanding the pathogen’s genetic diversity and population structure is crucial for developing and implementing novel and durable management strategies (Siah et al., 2018). Knowledge of the pathogen’s population structure can enhance predictions of the effectiveness and durability of host resistance. Plant pathogen populations with high genetic variability are more likely to adapt to resistant cultivars than populations with low genetic variability. Understanding the evolutionary forces controlling pathogen populations therefore holds enormous potential for the development and implementation of robust disease management measures (McDonald et al., 1995, 2015).

The advent of molecular marker technology has promising applications in plant pathology, enabling in assessing genetic variation among pathogens (Ramesh et al., 2020) and fingerprinting pathogen ecology and epidemiology. Internal transcribed spacer (ITS) sequences within ribosomal RNA genes (rDNA) are widely used as molecular probes to identify fungal pathogens (Yang et al., 2018). It is the prime fungal default region for species identification, and it can be retrieved using diagnostic primers that target the sequence (Das and Deb, 2015). However, research efforts to identify *Z. tritici* populations in Ethiopia based on ITS region sequencing are greatly lacking. Moreover, information on the genetic structure of the pathogen populations in the central and south-eastern parts of the country is limited. Simple sequence repeat (SSR) is the marker of choice and widely used to disclose the genetic structure of *Z. tritici* populations in various parts of the world including Tunisia (Boukef et al., 2013), England (Owen et al., 1998) and France (El Chartouni et al., 2011; Saintenac et al., 2018). Therefore, the aim of this study was molecular identification and genetic analysis of *Z. tritici* populations recovered from central and south-eastern regions of Ethiopia.

## 2 Materials and methods

### 2.1 Study areas and sampling procedure

**Figure 1:**
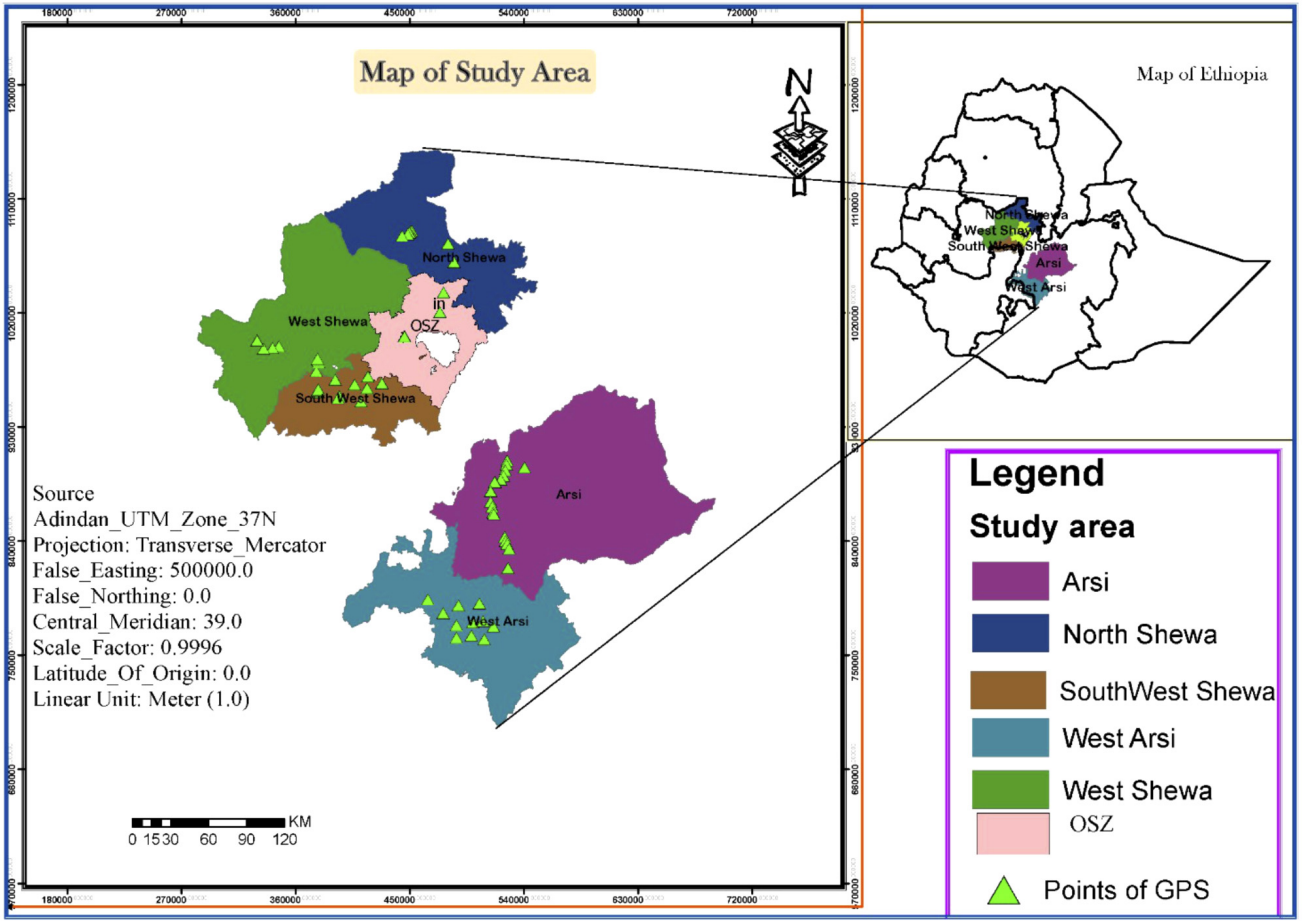
A map of Ethiopia showing the six administrative zones used for the collection of *Zymoseptoria tritici* isolates. GPS, global positioning system; OSZ, Oromia special Zone Surrounding Finfinne.

*Zymoseptoria tritici* isolates were recovered from STB-infected wheat leaf samples collected from six major wheat-growing zones in Ethiopia: West Shewa (WSH), Southwest Shewa (SWSH), North Shewa (NSH), Oromia special zone surrounding Finfinnee (OSZ), West Arsi (WA) and Arsi zone (AZ). These regions together represent 75% of the country’s total wheat production area. Samples were collected during the main cropping seasons (summer) of 2021, by following the main roads and accessible routes in a randomly selected major wheat-growing district. Stops were made at 5 – 10 km intervals where wheat fields were accessible, based on vehicle odometers. **Figure 1** and **Table 1** provide a map of the sample collection sites and summary information regarding the six *Z. tritici* populations. During collection, green leaves with symptoms of STB were collected in paper envelopes labelled with the sample code. The collected samples were then air-dried at room temperature and stored at 5 °C until isolation. The passport of the isolates used in this study and the Global Positioning System (GPS) data showing the six administrative zones covered for Z. tritici isolates collection were shown on **Supplementary Table 1**.

**Table 1:** Summary information for the six populations of *Zymoseptoria tritici* sampled in Ethiopia.

Population	Isolate code	Geographical position (UTM)		Elevation (m)
		Latitude range	Longitude range	
Arsi	ZTET043 – ZTET119	07° 47′ 775–08°17′ 474	039°12′ 465–039°26′ 206	1672–2856
North Shewa	ZTET179 – ZTET200	09°58′ 761–09°81′ 214	038°48′ 647–038°86′ 122	2550–3101
SW Shewa	ZTET145 – ZTET160	08°617′289–08°74′708	037°87′918–038°04′177	2259–2853
West Arsi	ZTET120 – ZTET144	07°01′438–08°12′959	038°78′611–039°36′766	1994–2713
West Shewa	ZTET161 – ZTET178	08°81′051–09°02′660	037°44′785–037°89′241	2230–3366
OSZ	ZTET001 – ZTET042	08°88′092–09°37′329	038°50′680–038°81′904	2158–2604

UTM, Universal Transverse Mercator coordinate system; SW Shewa, Southwest Shewa; OSZ, Oromia special zone surrounding Finfinnee.

### 2.2 Fungal isolation

Spore isolation and subsequent laboratory activities were carried out at the National Agricultural Biotechnology Research Centre (NABRC), Holeta, located 29 km west of Addis Abeba, Ethiopia. Fungal isolation was conducted following the procedure of Eyal and Levy (1987), with some modifications. Symptomatic leaf samples were cut into about 10cm lengths and placed on sterile filter paper in Petri dishes wetted with sterile distilled water. The Petri dishes were then placed in polyethylene plastic bags and incubated for 3–4 h at 24 °C. The samples were periodically checked under a stereoscopic dissecting microscope for the formation of a cloudy ooze on the top of the pycnidia. Using a flame-sterilized fine needle, the mono-pycnidia oozing drops were transferred onto potato dextrose agar (PDA; potato 200 g/l, dextrose 20 g/l, agar 15 g/l, Sigma-Aldrich, Hamburg, Germany). Colony morphology was confirmed under a microscope (40× objective, using a Konsortiet Laborlux 11 microscope; Leitz Wetzlar, Vienna, Germany), and creamy pink-colored colonies were streaked onto new PDA plates supplemented with 250 mg chloramphenicol. Inoculated Petri dishes were kept at 24 °C for 7–10 days until fungal growth was observed. Single spore-derived colonies were transferred into a liquid medium composed of 1% (w/v) yeast extract powder + 1% (w/v) sucrose, and the cultures were maintained on an orbital shaker at 130 rpm for three weeks, for spore multiplication. The fungal spores were then collected into Eppendorf tubes for molecular diversity analysis. In total, 200 single-spore-derived isolates were successfully retrieved and preserved in 25% glycerol at –80°C for further study.

### 2.3 Molecular identification

DNA extraction was carried out at the Swedish University of Agricultural Science (SLU), Department of Plant Breeding, Alnarp, Sweden. The glycerol-preserved fungal cultures were shipped to SLU and transferred on arrival to PDA (Sigma-Aldrich, Hamburg, Germany). Ten-day-old colonies grown on PDA were used for DNA isolation. DNA extraction was done using a Quick-DNA™ fungal/bacterial miniprep kit following the manufacturer’s protocol (Zymo Research, Irvine, CA, USA). The extracted DNA quality was checked by loading 5 µl DNA + 2 µl 6× loading dye with gel red on a 1% agarose gel and run at 100 V for 40 min. The DNA concentration was checked using a Nano-drop spectrophotometer (DS-11 Series Spectrophotometer/Fluorometer; De Novix, Wilmington, DE, USA). The isolates were confirmed using a universal primer (ITS5 forward and ITS4 reverse) (White et al., 1990) (**Table 2**) that targeted ITS sequences of rDNA. A polymerase chain reaction (PCR) was carried out in a 46-μl final reaction volume composed of 22 μl phire™ plant direct master mix, 1 μl each of the forward and reverse primers, 21 μl nuclease-free water and 1 μl genomic DNA, using a Bio-Rad Thermal cycler (Bio-Rad, Hercules, CA, USA). The PCR program followed an initial denaturation at 98 °C for 5 min followed by 40 cycles of 5 s denaturation at 98 °C, 1 min annealing at 55 °C, primer extension at 72 °C for 1 min, and a final extension step of 10 min at 72 °C. The PCR product was run on 2% agarose gel electrophoresis. A 1 kb marker was used to estimate the product size. The PCR products were then purified using a Quick-DNA™ fungal/bacterial miniprep purification kit (Zymo Research, Irvine, CA, USA) following the manufacturer’s protocol. The purified product quality was checked using Nano-drop spectrophotometers (DS-11 Series Spectrophotometer/Fluorometer; De Novix, Wilmington, DE, USA) and sent for Sanger sequencing (Eurofins Genomics, Ebensberg, Germany). DNA of five isolates failed the quality control, and hence, didn’t sequence. Thus, the sequence results of 195 isolates were blasted [using the Basic Local Alignment Search Tool (BLAST), nih.gov] in National Center for Biotechnology Information (NCBI), and a phylogenetic tree was constructed by using Fast Tree (Price et al., 2009). A Newick format tree was annotated using ITOL v6 (Letunic and Bork, 2024).

**Table 2:** Primer sequences for amplification of the internal transcribed spacer (ITS) for the molecular identification of *Zymoseptoria tritici*.

Locus	Primer	Orientation	Sequence (5′ to 3′)	Length	Reference
ITS	ITS5	Forward	TCCTCCGCTTA TTGATATGC	760 bp	White et al. (1990)
ITS	ITS4	Reverse	GGAAGTAAAAGTCGTAACAAGG		

### 2.4 Genetic structures using SSR markers

#### 2.4.1 Genotyping

The genetic structure of 200 isolates was explored using polymorphic published microsatellite markers. Twelve simple sequence repeat (SSR) markers (**Table 3**) were selected based on good amplification, polymorphism, specificity and suitability for multiplexing with DNA samples. The forward primers were 5′-labeled with four different fluorophore fluorescent dyes: FAM™, TAM™, ROX™ and HEX™ (Eurofins Genomics Ebensberg, Germany).

**Table 3:** The 12 simple sequence repeat (SSR) markers used for genetic diversity analyses of *Zymoseptoria tritici*.

Locus	Primer sequence (5' to 3')	Primer sequence (5′ to 3′)	Dye	Annealing temp. (°C)	Range of fragment size
ST3B	AAGAATCCCACCACCCAAAC	CACACGGCTCCTTTGACAC	HEX	68	263–299
ST2	ACACCAAAGAAGGATCCACG	GCCGGAGGTCTATCAGTTTG	HEX	68	338–365
ST3A	ACTTGGGGAGGTGTTGTGAG	ACGAATTGTTCATTCCAGCG	ROX	55	226–258
ST10	TCCGTCATCAACAACACCAG	TGGCCGTAGAACTGCTGAG	HEX	56	139–160
ST4	TGAACATCAACCTCACACGC	AGAAGAGGACGACCCACGAG	FAM	68	182–206
ST6	TCAATTGCCAATAATTCGGG	AGACGAGGCAGTTGGTTGAG	TAM	60	161–179
ST12	GAATCCACCTCTTCCTTGCC	AGGAGGATATCAAGGCCCAG	ROX	60	226–232
ST7	CACCACACCGTCGTTCAAG	CGTAAGTTGGTGGAGATGGG	FAM	63	171–227
ST9	CACCTCACTCCTCAATTCCG	GAAAGGTTGGTGTCGTGTCC	ROX	63	336–348
ST3C	TCCTATCAACTCCCGAGACG	CCGCACGTAGGAATTTTCAG	TAM	69	229–253
ST1	AATCGACCCCTTCCTTCAAC	GGGGGAGAGGCATAGTCTTG	TAM	69	192–222
ST5	GATACCAAGGTGGCCAAGG	CACGTTGGGAGTGTCGAAG	FAM	69	232–256

The PCR was performed in a total volume of 12.5 μl containing 6.75 μl phire™ plant direct master mix, 3.75 μl nuclease free water, 0.5 μl each of the forward and reverse primers, and 1 μl genomic DNA. A S1000™ Thermal Cycler (Bio-Rad) was used for amplification of the target loci. The primers’ annealing temperatures were gradient-optimized. The PCR program followed an initial denaturation at 95 °C for 5 min followed by 35 cycles of denaturation at 95 °C for 1 min, primer annealing at optimized temperatures of 55 °C, 56 °C, 60 °C, 63 °C and 69 °C for 1 min (**Table 3**), and primer extension at 72 °C. for 2 min, followed by a final extension at 72 °C for 10 min and holding at 4 °C. PCR amplification was verified before capillary electrophoresis by running 5 µl of the products on a 1.5% agarose gel with Gel Red at 100V for 2 hr and visualizing the gel using a BioDoc-It™ Imaging System (Upland, CA, USA). Twelve primer pairs of PCR products were multiplexed into three panels, each of which held four PCR products. To prevent overlapping, there had to be at least an 80 bp size difference between the PCR products in each panel that was labelled with the same fluorescent dye. The capillary electrophoresis of the PCR products was performed denatured in form amide at 99 °C for 3 min, and analyzed using the SeqStudio™ 8 Flex Genetic Analyzer (Thermo Fisher Scientific, Waltham, MA, USA) at SLU. The example of the Calibration chart of the sample during capillary electrophoresis was shown on **Supplementary Figure 1**.

#### 2.4.2 Data analysis

Following capillary electrophoresis, peak identification at the suggested threshold intensity was conducted using the software Gene Marker version 3.0.1 (Soft Genetics, LLC, State College, Pennsylvania, USA) with default parameters. The GS600 size standard was used to determine the fragment size. Each peak was considered as an allele at a codominant locus, and each individual or pooled genotype at each locus was noted. Power marker version 3.25 (Liu and Muse, 2005) was used to calculate the locus diversity parameters, including polymorphic information content (PIC), major allele frequency (MAF), observed heterozygosity (Ho) and gene diversity [expected heterozygosity (He)] throughout the entire population. The software GenAlEx version 6.501 (Peakall and Smouse, 2012) was used to compute Nei’s gene diversity (H), the effective number of alleles (Ne), Shannon’s information index (I), allelic frequency and other population diversity indices, including the number of alleles (Na), the number of private alleles (NPA), and the percentage of polymorphic loci (PPL) across all loci for each population. A genetic differentiation test (PhiPT, with p-values across 999 bootstrap replications) and pairwise population genetic distances and gene flow were analyzed using the same software. The mafft aligner was used for multiple sequence alignment (Yamada et al., 2016), and Fast Tree was employed to construct the tree. The constructed Newick format tree was annotated using ITOL version 6 (Letunic and Bork, 2024). R package gg fortify was used for the principal component analysis (PCA). Arlequin version 3.5.2.2 was used to compute the analysis of molecular variance (AMOVA) and estimate the variance components (Excoffier and Lischer, 2010). R scripts within the gg plot R statistical package were used to generate a heatmap of pairwise Fixation Index (F_ST_) using the resulting sequences. The equation Nm (Haploid) = [(1/PhiPT) – 1]/2 was used to calculate the gene flow (Nm) across populations, where PhiPT stands for the variance between populations/total genetic variants. A genetic dissimilarity matrix was created (Nei, 1972) based on Neighbour Joining (NJ) and Nei’s standard genetic distance (DST, corrected), as well as the continuous Euclidian dissimilarity index.

STRUCTURE version 2.3.4 was used with a Bayesian model-based clustering technique to analyze the population structure and admixture trends (Pritchard et al., 2000). Data was gathered over 250,000 Markov Chain Monte Carlo (MCMC) replications for Population Cluster (K) = 1–10 using 20 iterations for each K, with a burn-in period of 100,000 utilized in each run to estimate the true number of population clusters (K). The web-based STRUCTURE HARVESTER version 0.6.92 was used to forecast the ideal K value using the simulation method developed by (Evanno et al., 2005). Using the Clumpak beta version, a bar plot for the ideal K was generated (Kopelman et al., 2015).

## 3 Results

### 3.1 Molecular identification of *Z. tritici*

**Figure 2:**
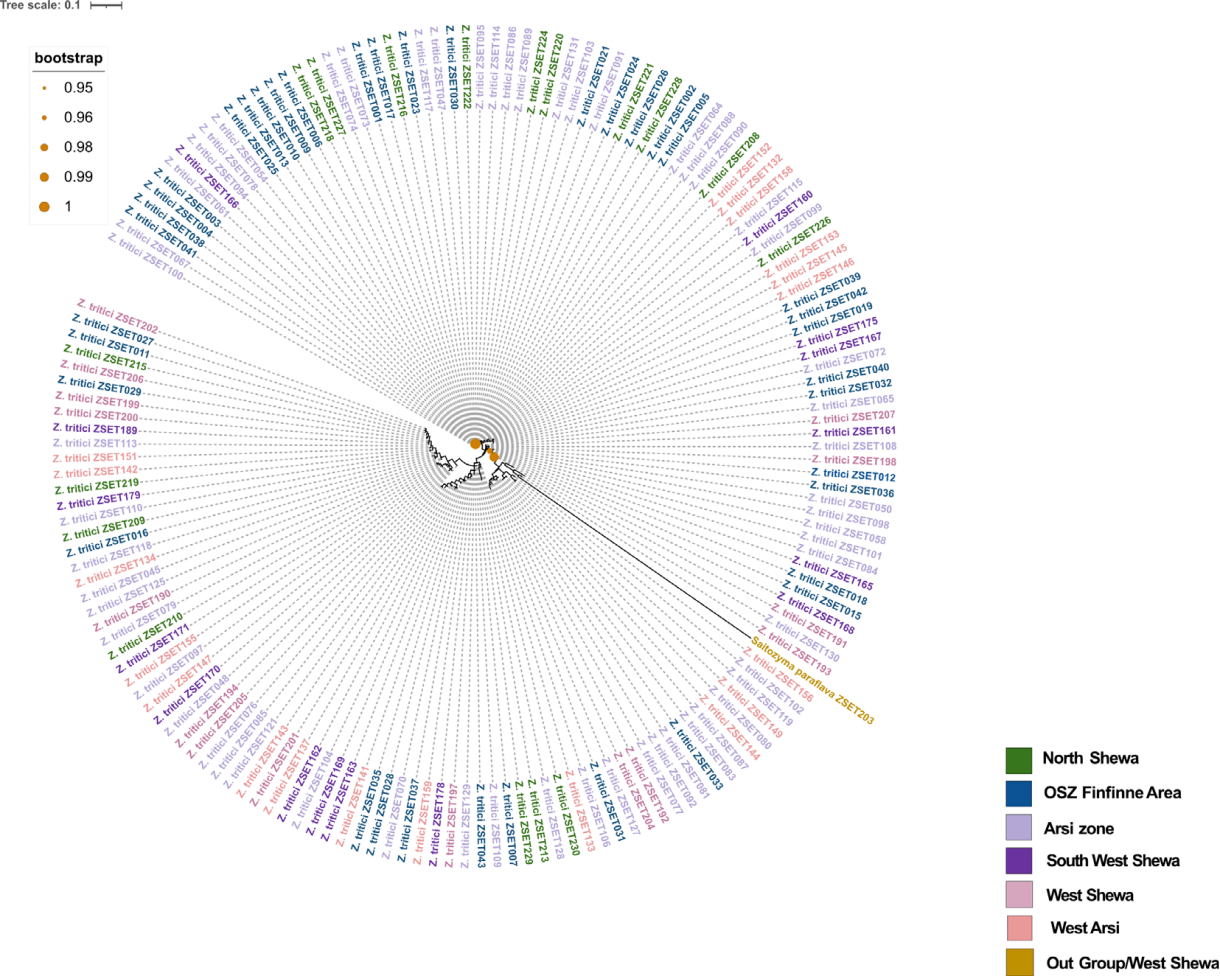
The phylogenetic tree of 167 *Zymoseptoria tritici* isolates collected from different regions (each color corresponds to each region) of Ethiopia. Tree was constructed using internal transcribed spacer (ITS) sequences and selected out group isolate is *Saitozyma paraflava*.

Out of 200 *Z. tritici* isolates sent for Sanger sequencing, 167 of them maintained the quality control and BLAST-searched NCBI levels needed to confirm fungal identity. A phylogenetic tree was then generated (**Figure 2**), revealing high levels of genetic admixture. The isolates did not cluster according to their geographical areas of sampling.

### 3.2 Genetic structure analyses

#### 3.2.1 The SSR markers’ polymorphism

All 12 SSR loci were polymorphic (**Supplementary Figure 2**) and produced a total of 66 alleles with an average of 5.5 alleles per locus (**Table 4**). For each locus, the frequency of the most common allele was less than 0.95 or 0.99, confirming the high polymorphism of the markers. They were highly informative, with mean values for Na of 6.32 (range 3.83–10.83), Ne of 2.90 (range 1.90–4.89), I of 1.22 (range 0.78–1.8), H of 0.57 (range 0.44–0.73), genetic differentiation statistics by locus (Gst) of 0.01 (range 0.01–0.03) and Ho of 0.77 (range 0.64–0.91). The mean locus value for total expected heterozygosity (Ht) =0.81, Fixation index (F_ST_) =0.03 and Nei’s gene diversity (H) =0.62. (**Table 4**).

**Table 4:** Informativeness and other genetic diversity summary statistics for 12 microsatellite loci across six populations of *Z. tritici* populations in Ethiopia.

Locus	Na	Ne	I	H	Gst	Uh	Ho	Hs	Ht	F_ST_	Nm	PIC
ST5	5.33	2.42	1.05	0.52	0.01	0.54	0.70	0.80	0.83	0.03	7.187	0.59
ST10	6.17	2.54	1.16	0.57	0.01	0.59	0.90	0.81	0.83	0.03	8.658	0.58
ST1	3.83	1.90	0.78	0.44	0.02	0.45	0.87	0.83	0.85	0.03	7.797	0.42
ST12	5.33	2.39	1.08	0.55	0.02	0.57	0.72	0.79	0.83	0.04	6.271	0.56
ST4	4.67	2.14	0.92	0.45	0.02	0.47	0.65	0.74	0.77	0.04	5.513	0.50
ST2	10.83	4.89	1.81	0.73	0.03	0.76	0.69	0.75	0.78	0.05	5.135	0.76
ST6	6.33	2.38	1.06	0.48	0.02	0.50	0.92	0.83	0.84	0.02	14.708	0.51
ST3A	9.00	3.30	1.57	0.67	0.01	0.70	0.74	0.80	0.83	0.03	7.863	0.68
ST7	7.83	3.76	1.56	0.69	0.01	0.72	0.83	0.78	0.80	0.03	9.041	0.69
ST3B	5.83	2.99	1.25	0.61	0.01	0.64	0.78	0.81	0.83	0.03	8.135	0.58
ST3C	4.83	2.77	1.17	0.61	0.01	0.64	0.80	0.78	0.80	0.03	8.398	0.59
ST9	5.83	3.31	1.32	0.63	0.01	0.66	0.69	0.77	0.79	0.03	9.468	0.65
Mean	6.32	2.90	1.23	0.58	0.01	0.61	0.76	0.78	0.81	0.03	8.181	0.59

Na, Observed number of alleles; Ne, Effective number of alleles; I, Shannon’s information statistic; H, Nei’s gene diversity; Gst, Genetic differentiation statistics by locus; Uh, Unbiased heterozygosity; Nm, estimate of the number of migrants (gene flow) from Gst where Nm = 0.5(1 – Gst)/Gst; PIC, Polymorphic information content; Ho, Mean observed heterozygosity over K populations, where Ho = (Sum (No. of Hets/N))/k; whereas No. of Hets is number of heterozygotes individuals carrying two different alleles at genetic locus, N is number of individuals in the population and K is number of loci or genetic markers. Hs, Mean expected heterozygosity He over k pops, where Hs, (Sum (1 – Sum pi^2))/K, and pi, pop allele frequency; Ht, Total expected heterozygosity, where Ht, 1-Sum pai^2, and pai, average pop allele frequency; F_ST_, Inbreeding coefficient within subpopulations, relative to total genetic differentiation among populations, where F_ST_ = (Ht – Hs)/Ht.

The highest values of Na (10.83), Ne (4.89), H (0.73) and I (1.81) were obtained for locus ST2, while locus ST1 gave the lowest values for these diversity indices (**Table 4**). The highest (14.7) and lowest (5.13) value for Nm was detected by locus ST6 and ST2, respectively. Except for ST1, which was moderately informative (PIC = 0.42), all the microsatellite loci used were highly informative (PIC >0.5; markers with a PIC value between 0.25 and 0.5 were considered as moderately informative, less than 0.25 as less informative, and more than 0.5 as highly informative).

#### 3.2.2 Genetic variability within and among populations

Average within-population genetic diversity estimates were determined for population sizes ranging from 16 to 76. The populations showed a wide range of within-population genetic differences, with mean values for Ne of 2.89, I of 1.23, H of 0.58, unbiased heterozygosity (Uh) of 0.60, and PPL of 98.61% (**Table 5**). *Zymoseptoria tritici* isolates obtained from OSZ showed the highest Na (9.33) value, followed by North Shewa populations. The highest Ne, H, I and Uh values were observed in North Shewa populations, while the lowest Ne and H values were observed in West Shewa populations. The PPL ranged from 91.67% (from West Shewa) to 100% (from OSZ, Arsi, West Arsi and Southwest Shewa) (**Table 5**).

**Table 5:** Allelic pattern and diversity index for six *Z. tritici* populations.

Population	N	Na	Ne	I	H	Uh	PPL
OSZ	43	9.33	3.41	1.56	0.68	0.69	100
Arsi	76	7.25	2.27	1.11	0.53	0.54	100
West Arsi	26	6.67	3.04	1.33	0.62	0.64	100
SW Shewa	16	3.50	2.21	0.89	0.49	0.52	100
West Shewa	18	4.33	2.16	0.93	0.47	0.50	91.67
North Shewa	21	7.27	4.25	1.58	0.69	0.72	100
Mean	33.3	6.38	2.89	1.23	0.58	0.60	98.61

N, Sample size; Na, Observed number of alleles; Ne, Effective number of alleles; H, Nei’s gene diversity; I, Shannon’s information index; Uh, Unbiased heterozygosity; PPL, percentage of polymorphic loci. SW Shewa, Southwest Shewa; OSZ, Oromia special zone surrounding Finfinnee.

**Figure 3:**
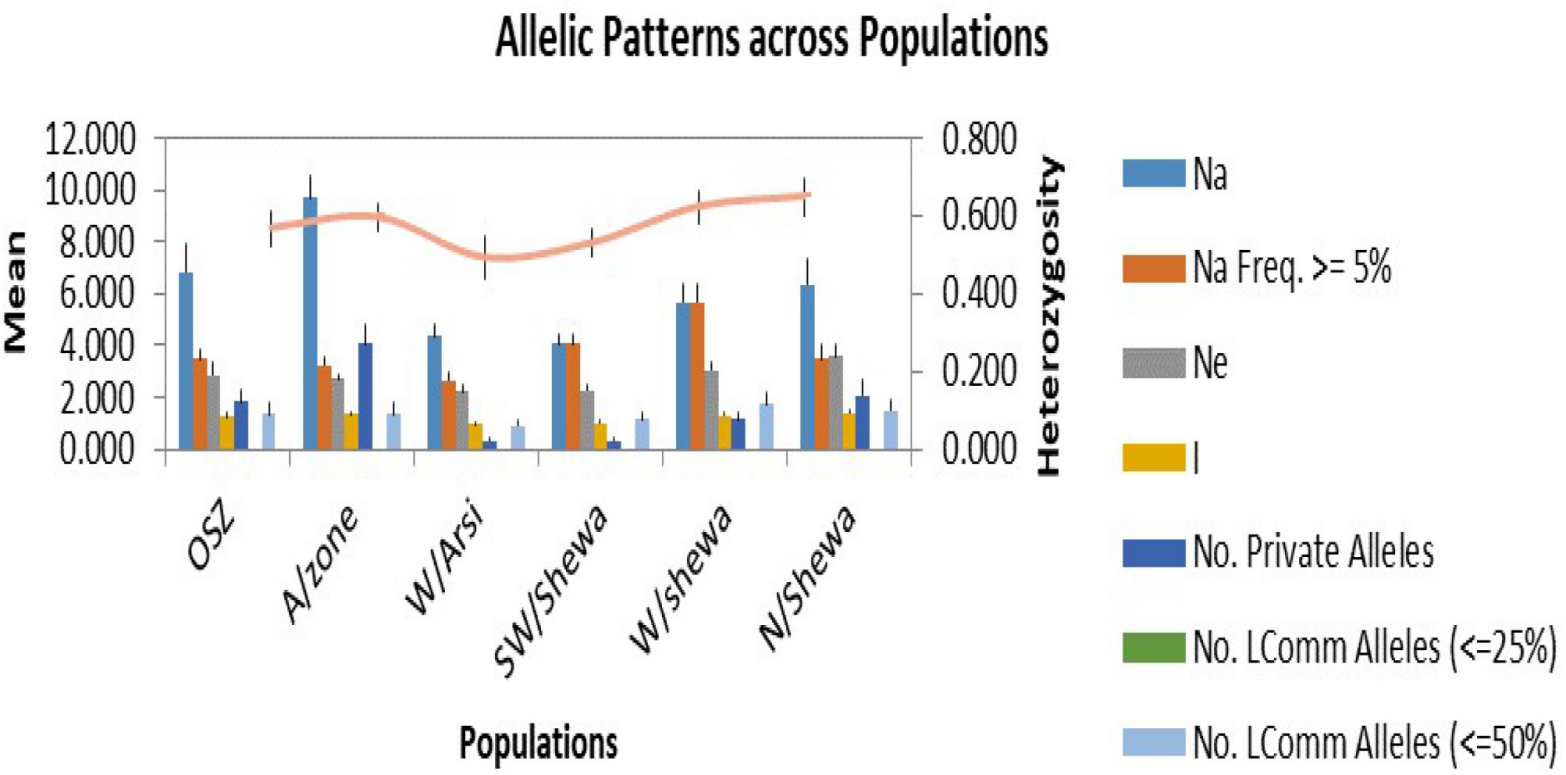
Allelic patterns across six populations of *Zymoseptoria tritici*. OSZ = Oromia special zone surrounding Finfinnee; A/Zone, Arsi; W/Arsi, West Arsi; SW/Shewa, Southwest Shewa; W/Shewa, West Shewa; N/Shewa, North Shewa. Na, numbers of different alleles; Na (Freq ≥5%), numbers of different alleles with a frequency of ≥5%; Ne, numbers of effective alleles; I, Shannon’s Information Index; No. Private Alleles, numbers of alleles unique to a single population; No. LComm Alleles (≤25%), numbers of locally common alleles (Freq. ≥ 5%) found in 25% or fewer populations and No. LComm Alleles (≤50%), numbers of locally common alleles (Freq. ≥ 5%) found in 50% or fewer populations. the orange line indicate expected heterozygosity.

In the allelic pattern analysis (**Figure 3**) of the 200 isolates, those obtained from Arsi showed the highest number of alleles (9.67), followed by OSZ and North Shewa, which scored 6.83 and 6.33 alleles, respectively. The SWSH *Z. tritici* population showed the Na of 4.08. The highest mean heterozygosity (0.64) was recorded for NHS isolates, followed by WSH and Arsi, with mean heterozygosity of 0.62 and 0.59, respectively. The effective number of alleles used for distinguishing locations from each other was highest for the North Shewa location and least (2.21) for the West Arsi location. Likewise, the highest (4.03) number of private alleles was observed in AZ, and the lowest (0.33) in *Z. tritici* populations of WA and SWSH (**Figure 3**).

#### 3.2.3 Genetic relationship within and among populations

An AMOVA revealed that 95% of the total genetic variation was attributable to within-populations variation, and therefore only 5% was accounted by the among-populations variation (**Table 6** and **Supplementary Figure 3**). The populations exhibited statistically significant but low genetic differentiation (F_ST_ = 0.02; p < 0.001).

**Table 6:** Analysis of molecular variance of 12 simple sequence repeats (SSR) from six Locations of *Zymoseptoria tritici*.

Source of variation	D.f.	Sum of squares	VC	Percentage of variation	FI	*P*-value
Among populations	5	50.99	Va = 0.09	2%	FIS =0.02	Va and FCT <0.001
Among individuals	194	941.45	Vb = 0.11	3%	FST = 0.02	Vb and FSC <0.001
Within individuals	200	928.00	Vc = 4.64	95%	FIT = 0.04	Vc and FST <0.001
Total	399	1920.44	4.83			
Gene flow (Nm = 14.7)						

Whereas d.f., degree of freedom; VC, Variance Component and FI, Fixation index; FIT is Overall inbreeding coefficient Calculated as = 1 – (HI/HT), FIS is Inbreeding coefficient calculated as = 1 – (HI/HS) FST = 1 – (HS/HT), Va, variance component among population; Vb, Variance component among individual; Vc, Variance component within individuals.

#### 3.2.4 Genetic relationships between the populations

**Table 7** shows the estimates for pairwise genetic distance and gene flow. The highest genetic distances of 0.30, 0.29 and 0.27 were observed between the populations of North Shewa and Southwest Shewa, North Shewa and Arsi, and North Shewa and West Shewa, respectively. Conversely, the lowest genetic distance (0.06) was observed between the *Z. tritici* populations of Southwest Shewa and Arsi, and West Shewa and Arsi (**Table 7**).

**Table 7:** Pairwise Nei’s genetic distance (H) (below diagonal) and gene flow (Nm) (Haploid) values (above diagonal) among six *Zymoseptoria tritici* populations of Ethiopia.

Population	OSZ	Arsi	West Arsi	SW Shewa	West Shewa	North Shewa
OSZ	–	9.00	16.50	6.32	5.95	9.21
Arsi	0.14	–	22.90	26.80	13.62	6.96
West Arsi	0.13	0.08	–	11.00	10.78	18.71
SW Shewa	0.18	0.06	0.10	–	25.90	7.95
West Shewa	0.16	0.06	0.11	0.08	–	8.51
North Shewa	0.19	0.29	0.19	0.30	0.27	–

SW Shewa, Southwest Shewa; OSZ, Oromia special zone surrounding Finfinnee.

**Figure 4:**
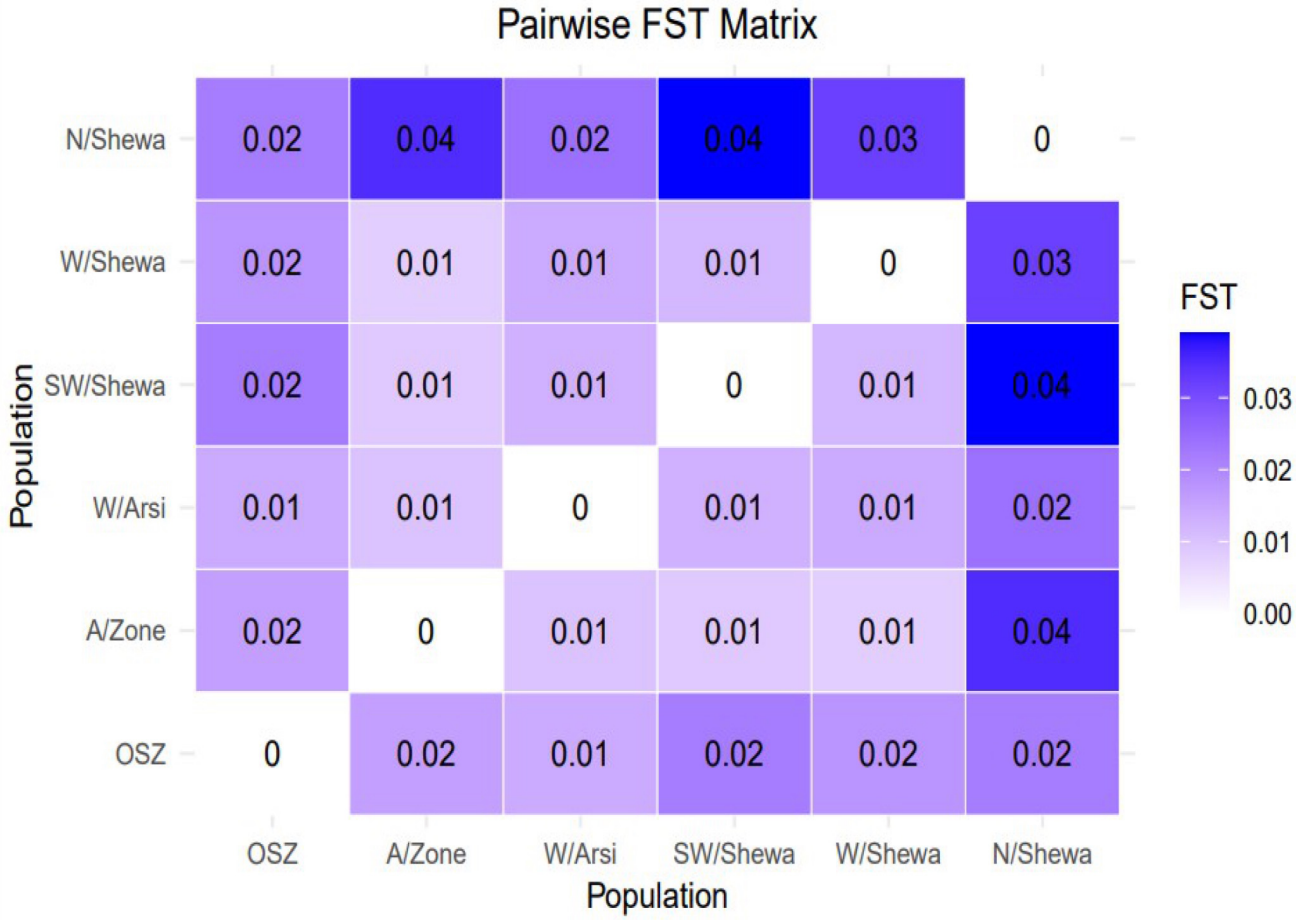
A graphical representation of a pairwise F_ST_ matrix for six populations of *Zymoseptoria tritici*. Whereas OSZ, Oromia special Zone surrounding Finfinne.

The pairwise coefficient of genetic differentiation between the populations ranged from 0.002 (between the populations of West Shewa and Southwest Shewa) to 0.077 (between West Shewa and OSZ). The highest and statistically significant genetic differentiation (PhiPT = 0.077, *p* < 0.001), with a gene flow rate of 5.9, was observed between the *Z. tritici* populations of West Shewa and OSZ, implying a relatively low gene flow between them. The second-highest genetic differentiation, with a gene flow rate of 6.33, was observed between the populations of Southwest Shewa and OSZ. The genetic differences between *Z. tritici* populations of Arsi and West Arsi, Arsi and Southwest Shewa, Southwest Shewa and West Shewa, and West Arsi and North Shewa, were not statistically significant (*p* > 0.05) (**Table 8**), whereas the corresponding gene flow for these pairs of populations was high (**Table 7**). The fixation index between the populations was very low, with the highest fixation index (F_ST_ = 0.04) observed between the populations of Southwest Shewa and North Shewa (**Figure 4**).

**Table 8:** Population genetic differentiation as measured by PhiPT (a genetic differentiation test for the variance between populations/total genetic variants) (below the diagonal) among six *Zymoseptoria tritici* populations from Ethiopia, with *p*-values above the diagonal.

Population	OSZ	Arsi	West Arsi	SW Shewa	West Shewa	North Shewa
OSZ	–	0.001	0.004	0.001	0.001	0.001
Arsi	0.05	–	0.010	0.057	0.001	0.001
West Arsi	0.03	0.02	–	0.014	0.003	0.023
SW Shewa	0.073	0.02	0.04	–	0.402	0.003
W/Shewa	0.08	0.04	0.04	0.00	–	0.004
N/Shewa	0.05	0.07	0.03	0.06	0.05	–

SW Shewa, South West Shewa; W/Shewa, West Shewa; N/Shewa, North Shewa; OSZ, Oromia special zone surrounding Finfinnee. 999 was number of Permutations used.

#### 3.2.5 Cluster analysis

**Figure 5:**
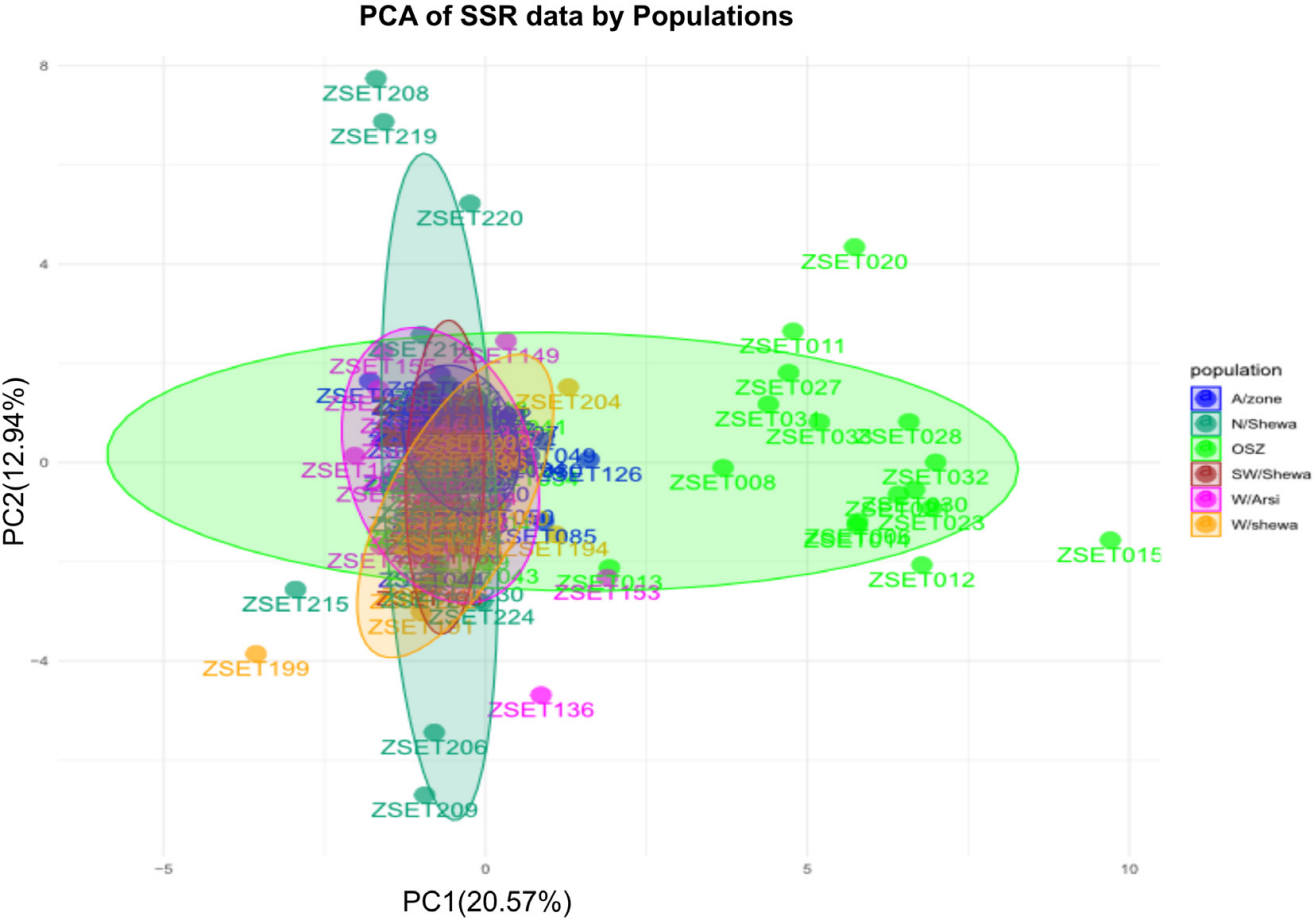
A principal component analysis (PCA) for the 200 individual *Zymoseptoria* tritici isolates as revealed by 12 simple sequence repeat (SSR) markers. Samples coded with the same symbol and color belong to the same population. OSZ, Oromia special Zone.

In the current study, principal component 1 (PC1) and principal component 2 (PC2) explained 33.51% (PC1 = 20.57% and PC2 = 12.94%) of the total genetic variation (**Figure 5**). PCA clustered the entire population into two subgroups with varying degrees of genetic admixture. The populations of Arsi, West Arsi, North Shewa, West Shewa and Southwest Shewa were clustered together. In contrast, individuals from OSZ showed a nearly uniform distribution on the two-dimensional coordinate plane, with little genetic admixture with the other *Z. tritici* populations (**Figure 5**). The finding indicated the presence of a significant gene flow between the geographical areas, resulting in poor clustering of the isolates. None of the clusters was composed of entirely isolates from a particular population (**Figure 5**).

**Figure 6:**
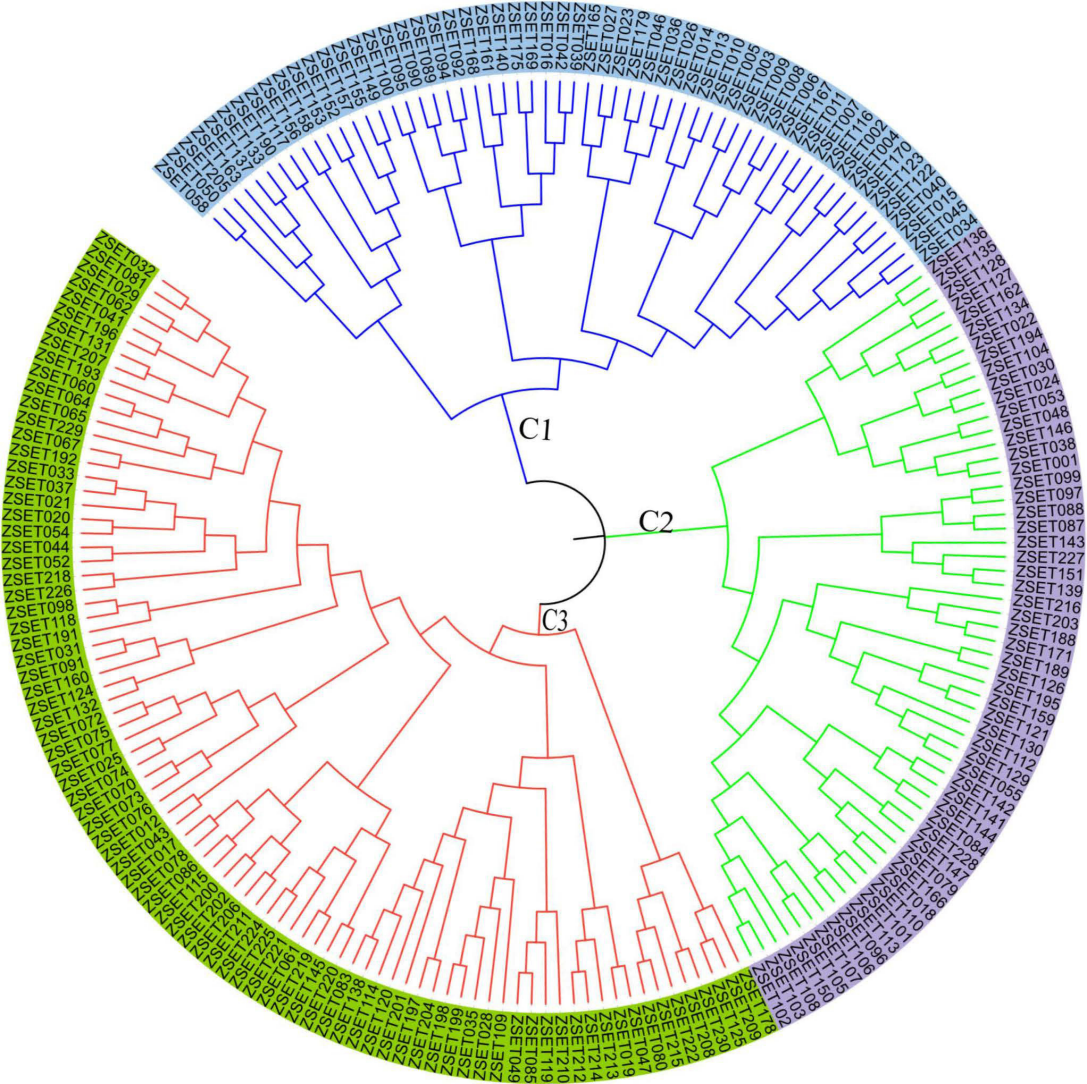
*The neighbor joining-based clustering of 200 Zymoseptoria tritici* isolates representing six populations. Samples coded with the same color belong to the same clade (C1–C3).

Accordingly, six populations of *Z. tritici* were sorted into three primary clusters (C1, C2 and C3), each of which was further divided into two sub-clusters based on an unweighted pair group method with arithmetic mean (UPGMA) of the measured of dissimilarity (**Figure 6**). Of the 200 isolates, 86 individuals (43%) belonged to C1, followed by C2 and C3, each of which had 57 (28.5%) individuals. None of the major clusters was made up of members of a single population, indicating high levels of gene flow. The UPGMA yielded a dendrogram that also divided the six populations into three main clusters (**Figure 6**).

#### 3.2.6 Population structure analysis

**Figure 7:**
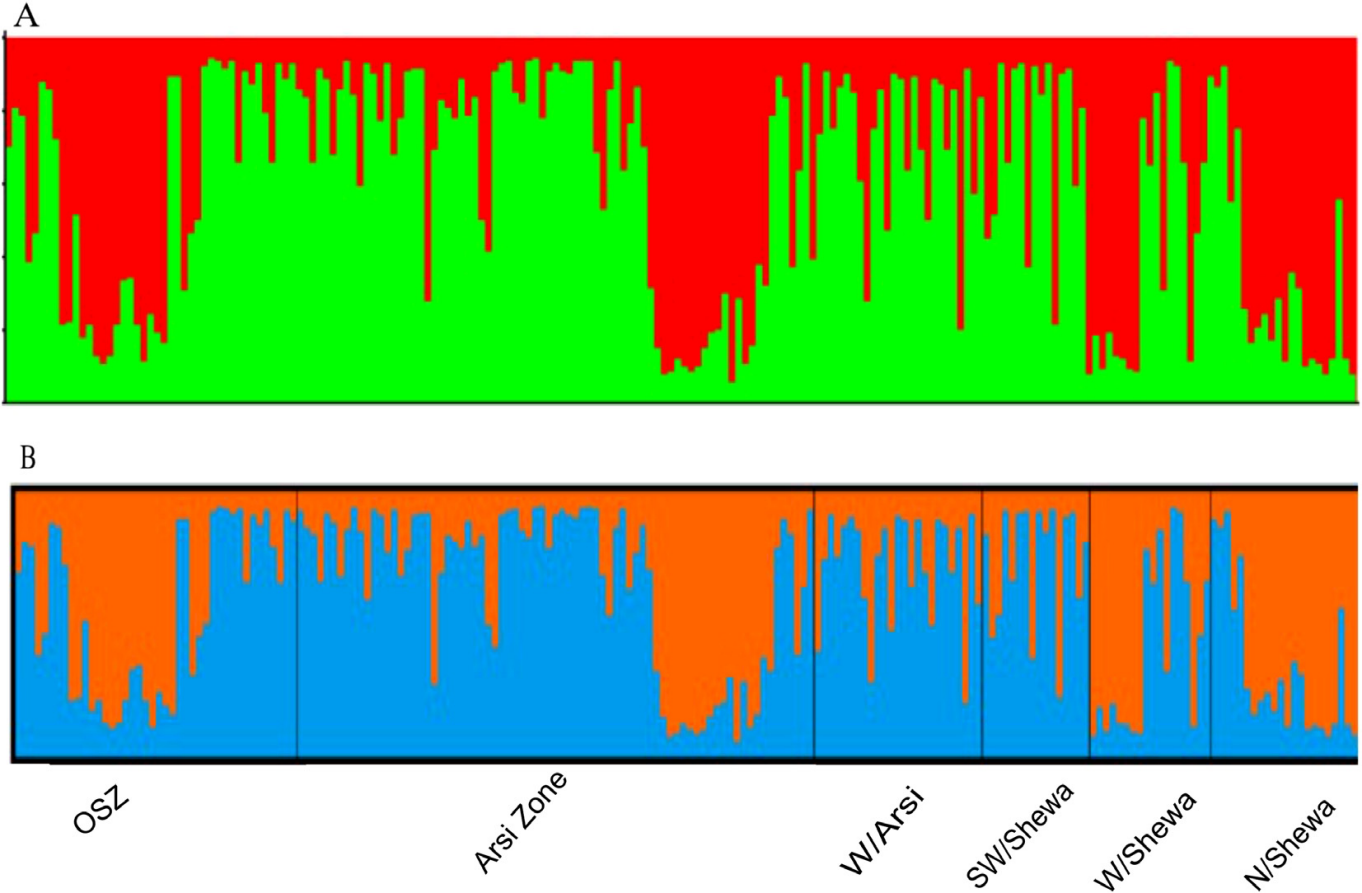
The population genetic structure of 200 individual isolates of *Zymoseptoria tritici* for K = 2. Each color represents a different cluster, and the different colors of each genotype represent membership in the different genetic populations. **(A)** Graphical representation of individual genotypes arranged according to the level of their membership in different clusters at 65% membership; **(B)** graphical display of the genetic structure of each population [isolates 43, 76, 26, 16, 18 and 21 representing OSZ (Oromia special zone surrounding Finfinnee), Arsi, West Arsi, SW/Shewa (Southwest Shewa) and N/Shewa (North Shewa), respectively].

The population structure analysis identified two genetic groups (K = 2), indicating that the isolates were sourced from two subpopulations (**Supplementary Figure 4**). None of the study populations was composed of exclusively isolates from a particular subpopulation. Each isolate shared alleles from both subpopulations, indicating the presence of genetic admixture (**Supplementary Figure 4**). Population structure analysis of the 200 *Z. tritici* isolates identified the best delta K value as 2 (**Figure 7**; the red and green colors represent the proposed isolates the two genetic groups shared).

## 4 Discussion

*Zymoseptoria tritici* is a haploid organism, and the SSR markers used in this study produced a single allele per isolate at each locus, confirming that they were present as single copies. The most common allele frequency at each locus was less than 0.95 or 0.99, confirming the loci as polymorphic, and thus a useful genetic tool for population genetics studies. There were three to seven alleles per locus, with an average of 5.5 alleles among the 12 loci. The average number of alleles observed in the present study was significantly higher than that reported by Mekonnen et al. (2020), who described an average of 3.5 alleles per locus among 182 isolates of *Z. tritici* of Ethiopia and the level reported by Siah et al. (2018), who described an average of 4.21 alleles per locus for *Z. tritici* populations of northern France using eight SSR markers.

All the SSR microsatellites used were highly polymorphic and informative (PIC = 0.59), indicating their relevance to unlocking the genetic structure of the target pathogen. They can therefore serve as a useful genetic tool to profile the genetic diversity of *Z. tritici* populations so as to develop and deploy durable and effective management strategies to control STB. The SSR markers used in this study showed a high locus diversity (H = 0.58, Nm =8.18 and PIC 0.59), which is in agreement with the report of Mekonnen et al.(2020), who observed a mean locus genetic diversity of 0.45, gene flow of 2.64 and PIC of 0.49 for 182 *Z. tritici* isolates also from Ethiopia.

The presence of high within-population genetic diversity in the *Z. tritici* populations was confirmed, with mean values for Na, Ne, I, He, H and PPL of 6.32, 2.90, 1.22, 0.58, 0.57 and 98.6%, respectively. The *Z. tritici* populations of Ethiopia appear to have higher within-population diversity than the *Z. tritici* populations of Tunisia that showed Ne = 1.67, I= 0.49, H = 0.28 and %PPL = 62.5 for 162 isolates obtained from three regions (Chedli et al., 2022). Higher genetic diversity was also reported for *Z. tritici* populations of United States (Gurung et al., 2011), Tunisia (Boukef et al., 2012) and northern France (L. El Chartouni et al., 2011). In the present study, the highest genetic diversity was observed in the *Z. tritici* populations collected from OSZ, followed by North Shewa and then West Arsi and Arsi, confirming these areas are ideal for studies of the pathogen’s genetics and genome, host–pathogen interactions, and also multi-location wheat germplasm screening for STB resistance.

The AMOVA test revealed the presence of greater genetic diversity (95%) within populations than between populations (5%). The higher within-population variation could be the result of random changes in the sequences of genes in the DNA (mutation) leading to the formation of new genes or alleles, random mating, and sexual reproduction resulting in the formation of new gene combinations. Higher within-population genetic diversity for *Z. tritici* was also reported elsewhere in the world including in Iran (Mahboubi et al., 2022), northern France (Siah et al., 2018) and Tunisia (Chedli et al., 2022). Hence, the significant level of genetic variability within the *Z. tritici* populations in the current study may be explained by spontaneous mutation and sexual recombination (McDonald and Linde, 2002).

The differentiation coefficient Fst is commonly used to assess population structure, with Fst values between 0.00 and 0.05 indicating minimal divergence, and Fst values between 0.05 and 0.15 indicating moderate divergence. The *Z. tritici* populations of OSZ and Southwest Shewa, OSZ and West Shewa, North Shewa and Arsi, and North Shewa and Southwest Shewa showed moderate divergence (Fst = 0.05–0.15), while all the other population pairs showed little population divergence (Fst <0.05).

The *Z. tritici* populations had high level of gene flow (Nm =14.7), resulting in lower population differentiation (FST = 0.01) (Chedli et al., 2022). Gene flow in *Z. tritici* occurs primarily through three mechanisms: the establishment of a sexual reproductive cycle, the existence of intermediate hosts such as some grasses, which can act as a green bridge, and the human transport of infected seeds and straw. Ascospores of *Z. tritici* may move across large distances by wind, which can lead to reduced genetic differentiation between populations (5%). Other factors that might have facilitated high gene flow among isolates from different geographical zones include the movement of plant parts such as straw, the exchange of infected seeds through trading, and long-distance movement of ascospores (McDonald and Mundt, 2016).

There was no clear, genetically identifiable clustering pattern in the *Z. tritici* populations studied. Neighbor joining-based cluster analyses did not group the populations according to their administrative zones, indicating the presence of close relationships and sharing of alleles among the different *Z. tritici* populations, which probably arose through long-distance movement of the spores by air and/or germplasm exchanges in the form of seeds from common market places. PCA is a technique frequently used in multivariate statistics to display patterns in genetic structure, and similarly to determine the amounts of variance described per component and cumulatively (Mekonnen et al., 2020). The *Zymoseptoria tritici* populations from different administrative zones showed significant genetic admixture, resulting in high gene flow (Nm =14.7), and hence PCA and STRUCTURE analyses failed to cluster the populations effectively based on the geographic areas of sampling. A Bayesian model-based clustering algorithm was used to determine the number of subpopulations (K) given an admixture model with associated allele frequencies. The STRUCTURE analysis also supported the PCA result, and the analysis revealed that the *Z. tritici* populations were sourced from two (K = 2) subpopulations, with a greater degree of genetic admixture among the *Z. tritici* isolates of the different administrative zones as a result of high gene flow. Similar population genetic admixtures was documented for *Z. tritici* populations in Tunisia (Chedli et al., 2022).

## 5 Conclusion

STB caused by *Z. tritici* (*M. graminicola*) represents a serious threat to global wheat production, and is a major bottleneck to wheat production in Ethiopia, causing significant yield loss. In order to develop and implement long-lasting and successful management strategies, accurate identification and data on the genetic structure of the pathogen populations gathered from hotspot sites are crucial. In this study, a total of 167 isolates of this fungus was successfully characterized by sequencing the ITS rDNA regions. Sequence-based phylogenetic analyses revealed a high level of genetic admixture among the *Z. tritici* isolates collected from various administrative zones. The genetic structure analysis of 200 *Z. tritici* isolates recovered from six administrative zones using 12 SSR also revealed presence of high levels of genetic diversity, with 95% of the total genetic variation residing within populations. Sexual recombination and spontaneous mutations could be the driving forces for the high within population’s variations. The high pathogen genetic diversity implies the need for broad spectrum resistance breeding, and also adoption of integrated disease management strategies. Among the six populations, those obtained from OSZ, Arsi, West Arsi and North Shewa showed the highest genetic diversity. These locations therefore provide hotspots for *Z. tritici* genetic analyses, and can serve as good environments for screening wheat germplasm for resistance to STB. The *Z. tritici* populations exhibited low to moderate pairwise genetic differentiation (Fst = 0.05–0.15), probably because of the high gene flow rate (Nm = 14.7) through long-distance movement of the spore. Consequently, clustering, PCA and STRUCTURE analyses failed to separate the populations into distinct groups based on their sampling areas. To confirm the present results, and to gain further insight into the pathogen’s genetic structure, we recommend continued analyses of the pathogen populations, including from other wheat major wheat-growing areas, as well as populations obtained from different years at different growth stages of the pathogen and also from different hosts both bread and durum wheat and alternative hosts.

## Data Availability

The datasets presented in this study can be found in online repositories. The names of the repository/repositories and accession number(s) can be found in the article/supplementary material.
