# Supplementary material for: Analysis of genetic diversity of Zymoseptoria tritici populations in central and south-eastern Ethiopia
Source: Front Plant Sci. 2025 Apr 9;16:1505455. doi: 10.3389/fpls.2025.1505455 (PMC12014633; doi:10.3389/fpls.2025.1505455)
Supplement: Supplementary file 1 [file DataSheet1.pdf]

Supplementary Table 1. Global Positioning System (GPS) data showing the six administrative zones covered for *Z. tritici* isolates collection.

NO	Sample ID	Population	Latitude (X - axis)	Longitude (Y-axis)	Altitude (A)
1	ZSET001	OSZ	09°05'69''	38°50'69''	2376
2	ZSET002	OSZ	09 ° 0597527	38 ° 5068122	2388
3	ZSET003	OSZ	09 ° 0597444	38 ° 5068011	2372
4	ZSET004	OSZ	09 ° 0596175	38 ° 5068292	2381
5	ZSET005	OSZ	09 ° 0596485	38 ° 5068793	2385
6	ZSET006	OSZ	09 ° 0595688	38 ° 5071029	2401
7	ZSET007	OSZ	09 ° 0596034	38 ° 5071266	2398
8	ZSET008	OSZ	09 ° 0596284	38 ° 5071872	2401
9	ZSET009	OSZ	09 ° 0596105	38 ° 5071828	2398
10	ZSET010	OSZ	09 ° 0594488	38 ° 5072152	2408
11	ZSET011	OSZ	09 ° 0594472	38 ° 5072374	2408
12	ZSET012	OSZ	09 ° 0594485	38 ° 5072549	2408
13	ZSET013	OSZ	09 ° 059435	38 ° 5072471	2407
14	ZSET014	OSZ	09 ° 0594342	38 ° 5072336	2406
15	ZSET015	OSZ	09 ° 0603669	38 ° 5073142	2420
16	ZSET016	OSZ	09 ° 0603775	38 ° 5073718	2419
17	ZSET017	OSZ	09 ° 0605835	38 ° 5075603	2430
18	ZSET018	OSZ	09 ° 0603903	38 ° 507583	2412
19	ZSET019	OSZ	09 ° 0605595	38 ° 5075624	2383
20	ZSET020	OSZ	09 ° 0605604	38 ° 5076252	2381
21	ZSET021	OSZ	09 ° 0605696	38 ° 5077458	2369
22	ZSET022	OSZ	09 ° 0605607	38 ° 5077651	2372
23	ZSET023	OSZ	09 ° 0605514	38 ° 5077176	2372
24	ZSET024	OSZ	09 ° 060557	38 ° 5077263	2373
25	ZSET025	OSZ	09 ° 0604949	38 ° 5078732	2469
26	ZSET026	OSZ	09 ° 0569553	38 ° 507404	2375
27	ZSET027	OSZ	09 ° 0569129	38 ° 5073957	2379

28	ZSET028	OSZ	09 ° 0569022	38 ° 5075294	2378
29	ZSET029	OSZ	09 ° 056879	38 ° 5074794	2382
30	ZSET030	OSZ	09 ° 056807	38 ° 5074122	2376
31	ZSET031	OSZ	09 ° 0567543	38 ° 5074043	2377
32	ZSET032	OSZ	09 ° 05654	38 ° 5072454	2383
33	ZSET033	OSZ	09 ° 0565292	38 ° 5072762	2382
34	ZSET034	OSZ	09 ° 0563627	38 ° 5071617	2367
35	ZSET035	OSZ	09 ° 056241	38 ° 5071142	2394
36	ZSET036	OSZ	09 ° 0562057	38 ° 5070435	2397
37	ZSET037	OSZ	09 ° 0564594	38 ° 5070058	2400
38	ZSET038	OSZ	09 ° 0564453	38 ° 507015	2397
39	ZSET039	OSZ	08 ° 9273379	38 ° 8190416	2202
40	ZSET040	OSZ	08 ° 9273469	38 ° 8190094	2206
41	ZSET041	OSZ	08 ° 9273442	38 ° 8189631	2204
42	ZSET042	OSZ	08 ° 8809197	38 ° 8163993	2157
43	ZSET043	OSZ	09 ° 0569269	38 ° 5069977	2159
44	ZSET044	A/zone	08 ° 1747366	39 ° 2432836	2133
45	ZSET045	A/zone	08 ° 1487019	39 ° 2368839	2118
46	ZSET046	A/zone	08 ° 1487761	39 ° 2367133	2082
47	ZSET047	A/zone	08 ° 1085819	39 ° 2282024	2026
48	ZSET047	A/zone	08 ° 064613	39 ° 2173333	2090
49	ZSET048	A/zone	08 ° 0415884	39 ° 1970149	2176
50	ZSET049	A/zone	08 ° 0201173	39 ° 1571817	2177
51	ZSET050	A/zone	08 ° 0201061	39 ° 1571715	2135
52	ZSET052	A/zone	08 ° 0203317	39 ° 1559175	1946
53	ZSET053	A/zone	08 ° 0207988	39 ° 1558163	1955
54	ZSET054	A/zone	08 ° 0206275	39 ° 155814	2011
55	ZSET055	A/zone	08 ° 0207357	39 ° 1557938	2027
56	ZSET057	A/zone	08 ° 0205833	39 ° 1558425	1785
57	ZSET058	A/zone	08 ° 0211456	39 ° 1563795	2069
58	ZSET060	A/zone	08 ° 0205748	39 ° 1561334	1916
59	ZSET061	A/zone	08 ° 0216847	39 ° 1547277	2009
60	ZSET062	A/zone	08 ° 0212884	39 ° 154875	1834

61	ZSET064	A/zone	08 ° 0217344	39 ° 1538325	2209
62	ZSET065	A/zone	08 ° 0207542	39 ° 1550355	1671
63	ZSET067	A/zone	08 ° 0220362	39 ° 1546981	2155
64	ZSET070	A/zone	08 ° 0209196	39 ° 154974	2089
65	ZSET072	A/zone	08 ° 0211844	39 ° 1546004	2194
66	ZSET073	A/zone	08 ° 0208715	39 ° 1549649	2130
67	ZSET074	A/zone	08 ° 0209997	39 ° 1549862	2090
68	ZSET075	A/zone	08 ° 0215224	39 ° 1546602	2201
69	ZSET076	A/zone	08 ° 0211649	39 ° 1546203	2336
70	ZSET077	A/zone	07 ° 9571685	39 ° 1265004	2521
71	ZSET079	A/zone	07 ° 8810894	39 ° 1246496	2530
72	ZSET079	A/zone	07 ° 88106	39 ° 124785	2513
73	ZSET080	A/zone	07 ° 8810691	39 ° 1249253	2535
74	ZSET082	A/zone	07 ° 8810242	39 ° 1251437	2537
75	ZSET083	A/zone	07 ° 8808577	39 ° 1251743	2606
76	ZSET084	A/zone	07 ° 8427269	39 ° 1380444	2607
77	ZSET085	A/zone	07 ° 8422822	39 ° 138437	2574
78	ZSET086	A/zone	07 ° 8028474	39 ° 1424028	2561
79	ZSET087	A/zone	07 ° 8028749	39 ° 142656	2580
80	ZSET088	A/zone	07 ° 8025738	39 ° 1427628	2568
81	ZSET089	A/zone	07 ° 8025157	39 ° 1427599	2566
82	ZSET090	A/zone	07 ° 7912048	39 ° 1488327	2562
83	ZSET091	A/zone	07 ° 7912829	39 ° 148794	2559
84	ZSET092	A/zone	07 ° 7912544	39 ° 1488595	2470
85	ZSET094	A/zone	07 ° 6295044	39 ° 224966	2471
86	ZSET095	A/zone	07 ° 6294857	39 ° 2249666	2589
87	ZSET096	A/zone	07 ° 5998192	39 ° 230454	2588
88	ZSET097	A/zone	07 ° 5998809	39 ° 2304643	2599
89	ZSET098	A/zone	07 ° 5997786	39 ° 2303337	2599
90	ZSET099	A/zone	07 ° 5997209	39 ° 2304392	2668
91	ZSET100	A/zone	07 ° 5797133	39 ° 2431636	2669
92	ZSET101	A/zone	07 ° 5797652	39 ° 2431495	2663
93	ZSET102	A/zone	07 ° 5799478	39 ° 2431251	2675

94	ZSET103	A/zone	07 ° 5799521	39 ° 2432951	2794
95	ZSET104	A/zone	07 ° 5437801	39 ° 2552885	2797
96	ZSET105	A/zone	07 ° 5438331	39 ° 2552222	2814
97	ZSET106	A/zone	07 ° 5442644	39 ° 255244	2797
98	ZSET107	A/zone	07 ° 5442459	39 ° 255319	2792
99	ZSET108	A/zone	07 ° 5446436	39 ° 25584	2793
100	ZSET109	A/zone	07 ° 5446458	39 ° 2558506	2784
101	ZSET110	A/zone	07 ° 544628	39 ° 2560705	2803
102	ZSET112	A/zone	07 ° 5448962	39 ° 2560249	2784
103	ZSET113	A/zone	07 ° 5447388	39 ° 2557504	2782
104	ZSET114	A/zone	07 ° 5447398	39 ° 255736	2788
105	ZSET115	A/zone	07 ° 5446688	39 ° 2557108	2791
106	ZSET116	A/zone	07 ° 5446858	39 ° 2557205	2789
107	ZSET117	A/zone	07 ° 5446853	39 ° 2556933	2791
108	ZSET118	A/zone	07 ° 5446774	39 ° 2556623	2793
109	ZSET119	A/zone	07 ° 5446602	39 ° 2556463	2845
110	ZSET120	A/zone	07 ° 5444003	39 ° 255155	2837
111	ZSET121	A/zone	07 ° 5444045	39 ° 2551809	2840
112	ZSET122	A/zone	07 ° 5443574	39 ° 2551551	2846
113	ZSET123	A/zone	07 ° 5443482	39 ° 255141	2839
114	ZSET124	A/zone	07 ° 5443438	39 ° 255157	2837
115	ZSET125	A/zone	07 ° 5443406	39 ° 2551559	2840
116	ZSET126	A/zone	07 ° 5443333	39 ° 2551352	2790
117	ZSET127	A/zone	07 ° 5441266	39 ° 2550439	2793
118	ZSET128	A/zone	07 ° 5441097	39 ° 2550609	2855
119	ZSET129	A/zone	07 ° 4777469	39 ° 2620539	2854
120	ZSET130	W/Arsi	07 ° 3715682	39 ° 2542695	2850
121	ZSET131	W/Arsi	07 ° 3714953	39 ° 2543007	2973
122	ZSET132	W/Arsi	07 ° 0837071	38 ° 7862882	2979
123	ZSET133	W/Arsi	07 ° 0837347	38 ° 7863517	2978
124	ZSET134	W/Arsi	07 ° 0834679	38 ° 786532	2932
125	ZSET136	W/Arsi	07 ° 083497	38 ° 7865181	2976
126	ZSET137	W/Arsi	07 ° 0835245	38 ° 7865191	2974

127	ZSET138	W/Arsi	07 ° 0835192	38 ° 7865106	2980
128	ZSET139	W/Arsi	07 ° 0835322	38 ° 7865187	2983
129	ZSET140	W/Arsi	07 ° 0835361	38 ° 7865125	2977
130	ZSET141	W/Arsi	07 ° 0835375	38 ° 7865091	2965
131	ZSET142	W/Arsi	07 ° 0835409	38 ° 7865042	2955
132	ZSET143	W/Arsi	07 ° 0839987	38 ° 786236	2628
133	ZSET144	W/Arsi	07 ° 0840209	38 ° 7862003	2634
134	ZSET145	W/Arsi	07 ° 0842813	38 ° 7861392	2709
135	ZSET146	W/Arsi	07 ° 0843064	38 ° 7861087	2707
136	ZSET147	W/Arsi	07 ° 0843233	38 ° 7861251	2708
137	ZSET148	W/Arsi	07 ° 0840605	38 ° 7863886	2711
138	ZSET149	W/Arsi	07 ° 0840369	38 ° 7865058	2708
139	ZSET150	W/Arsi	07 ° 0840395	38 ° 7865294	2712
140	ZSET151	W/Arsi	07 ° 0840259	38 ° 7865139	2710
141	ZSET152	W/Arsi	07 ° 084013	38 ° 7865099	2711
142	ZSET153	W/Arsi	07 ° 0838573	38 ° 7865496	2645
143	ZSET155	W/Arsi	07 ° 0843883	38 ° 7872663	2649
144	ZSET156	W/Arsi	07 ° 0202747	38 ° 9988112	2615
145	ZSET157	W/Arsi	07 ° 014381	39 ° 0288142	2616
146	ZSET158	SW/Shewa	08 ° 1224615	39 ° 2874394	2620
147	ZSET159	SW/Shewa	08 ° 1225958	39 ° 2873934	2652
148	ZSET160	SW/Shewa	08 ° 6891972	38 ° 2384404	2673
149	ZSET161	SW/Shewa	08 ° 6323209	38 ° 0417685	2596
150	ZSET162	SW/Shewa	08 ° 6323779	38 ° 0416194	2595
151	ZSET163	SW/Shewa	08 ° 6323652	38 ° 0417241	2594
152	ZSET165	SW/Shewa	08 ° 6323268	38 ° 041489	2589
153	ZSET166	SW/Shewa	08 ° 6323252	38 ° 0414472	2602
154	ZSET167	SW/Shewa	08 ° 6323454	38 ° 0414858	2531
155	ZSET168	SW/Shewa	08 ° 6324223	38 ° 0414516	2488
156	ZSET169	SW/Shewa	08 ° 6324054	38 ° 0414585	2230
157	ZSET170	SW/Shewa	08 ° 6318198	38 ° 0413837	1994
158	ZSET171	SW/Shewa	08 ° 6172886	38 ° 0316986	2162
159	ZSET175	SW/Shewa	08 ° 6173279	38 ° 0317713	2161

160	ZSET178	SW/Shewa	08° 6173265	38° 0317812	2139
161	ZSET179	SW/Shewa	08° 6173707	38° 031784	2379
162	ZSET188	W/shewa	08° 6598594	37° 8911229	2392
163	ZSET189	W/shewa	08° 6737157	37° 8838739	2386
164	ZSET190	W/shewa	08° 673814	37° 8838156	2402
165	ZSET191	W/shewa	08° 6736658	37° 8837858	2407
166	ZSET192	W/shewa	08° 6799812	37° 8882704	2409
167	ZSET193	W/shewa	08° 6798205	37° 8882773	2385
168	ZSET194	W/shewa	08° 6798384	37° 8883107	2387
169	ZSET195	W/shewa	08° 6801966	37° 8881866	2372
170	ZSET196	W/shewa	08° 7470752	37° 8791774	2305
171	ZSET197	W/shewa	08° 8105019	37° 874691	2347
172	ZSET198	W/shewa	08° 8721521	37° 8924064	2344
173	ZSET199	W/shewa	08° 8722338	37° 8922632	2307
174	ZSET200	W/shewa	08° 8722454	37° 8921219	2305
175	ZSET201	W/shewa	08° 8722	37° 8921166	2309
176	ZSET202	W/shewa	08° 8855991	37° 8889731	2258
177	ZSET203	W/shewa	08° 8978021	37° 8840987	2267
178	ZSET204	W/shewa	08° 8986769	37° 8834042	2322
179	ZSET205	W/shewa	08° 8983834	37° 8832602	2306
180	ZSET206	N/Shewa	09° 2336907	38° 7591871	2279
181	ZSET207	N/Shewa	09° 2336843	38° 7593731	2453
182	ZSET208	N/Shewa	09° 3732933	38° 7861876	2400
183	ZSET209	N/Shewa	09° 5876111	38° 8610995	2434
184	ZSET210	N/Shewa	09° 5876923	38° 8611255	2508
185	ZSET211	N/Shewa	09° 5876248	38° 8612242	2551
186	ZSET212	N/Shewa	09° 7200233	38° 8189879	2551
187	ZSET213	N/Shewa	09° 8121388	38° 5577201	2507
188	ZSET214	N/Shewa	09° 8119593	38° 5576232	2852
189	ZSET215	N/Shewa	09° 8118599	38° 5578467	3365
190	ZSET216	N/Shewa	09° 8118214	38° 5579527	2687
191	ZSET218	N/Shewa	09° 8117915	38° 5579083	2686
192	ZSET219	N/Shewa	09° 807808	38° 54955	2671

193	ZSET220	N/Shewa	09 ° 8077031	38 ° 5495874	2668
194	ZSET221	N/Shewa	09 ° 8076176	38 ° 5494552	2627
195	ZSET224	N/Shewa	09 ° 8076429	38 ° 5493765	2524
196	ZSET225	N/Shewa	09 ° 8012966	38 ° 5456616	2579
197	ZSET226	N/Shewa	09 ° 8014966	38 ° 5458269	2501
198	ZSET227	N/Shewa	09 ° 7976163	38 ° 5428452	2502
199	ZSET228	N/Shewa	09 ° 7976281	38 ° 5427114	2404
200	ZSET230	N/Shewa	09 ° 7978007	38 ° 5424305	2229

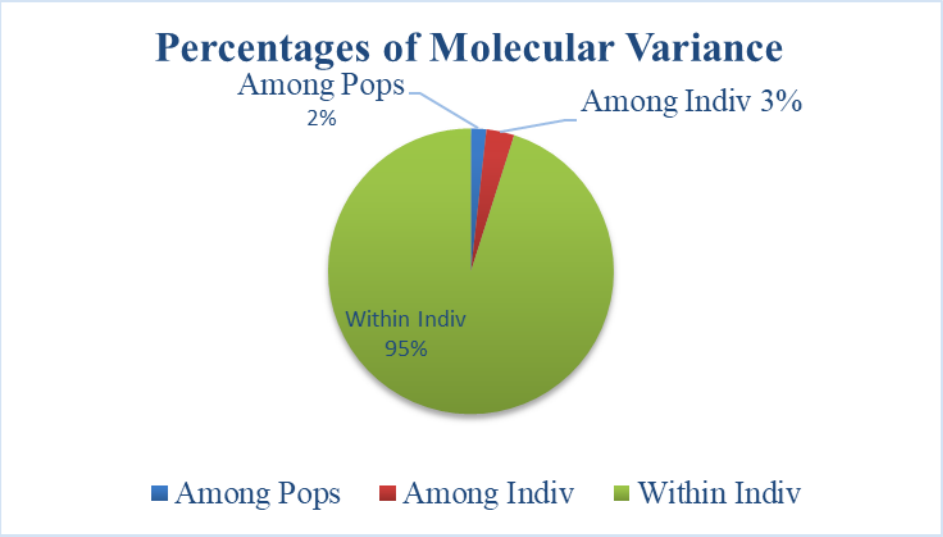

Supplementary Figure 1. Percentage of molecular variance among population and within population.

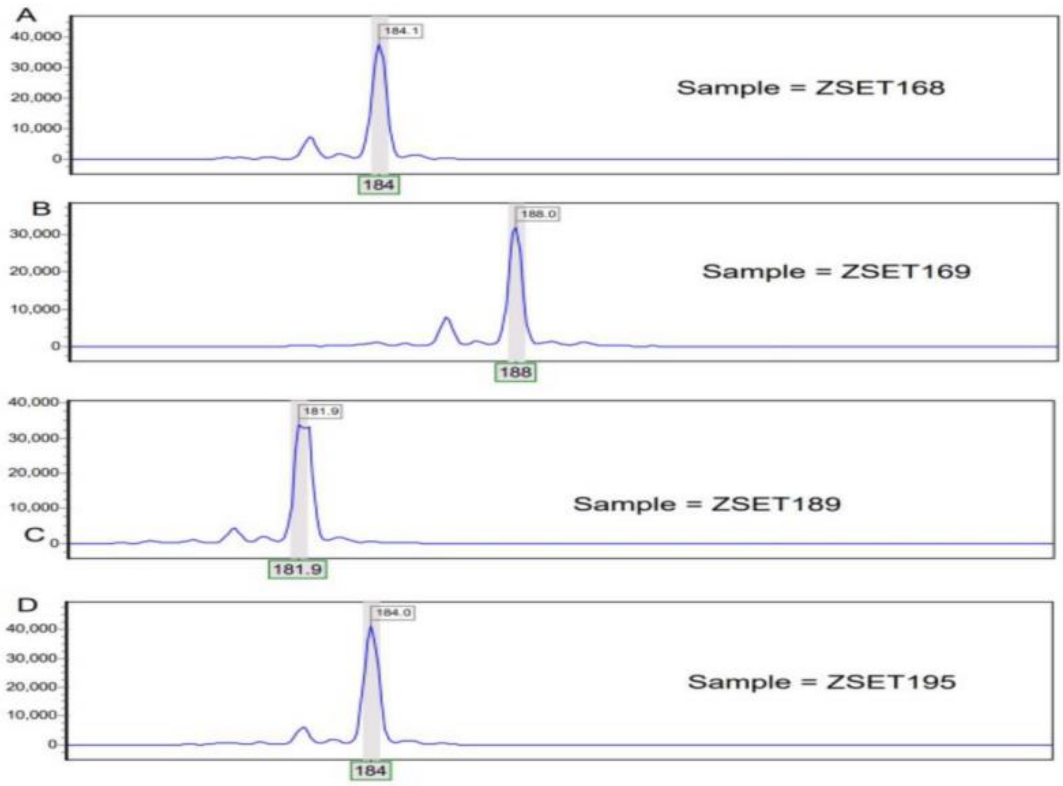

Supplementary Figure 2. Capillary Electrophoresis result or Electrophoretograms for four Zymoseptoria tritici isolates at locus ST2 showing different sizes (bp) of amplification: (A) 184 bp in isolate ZSET168, (B) 188 bp in ZSET169, (C) 182 bp in ZSET189, and (D) 184 bp in ZSET168.

No.	Name
1	A01_ZSET_ST2_ST3A_ST4_ST6_001_Sample_20230815_102436.fsa
2	B01_ZSET_ST2_ST3A_ST4_ST6_002_Sample_20230815_102437.fsa
3	D01_ZSET_ST2_ST3A_ST4_ST6_004_Sample_20230815_102439.fsa
4	H01_ZSET_ST2_ST3A_ST4_ST6_010_Sample_20230815_102443.fsa
5	G01_ZSET_ST2_ST3A_ST4_ST6_009_Sample_20230815_102442.fsa
6	C01_ZSET_ST2_ST3A_ST4_ST6_003_Sample_20230815_102438.fsa
7	E01_ZSET_ST2_ST3A_ST4_ST6_005_Sample_20230815_102440.fsa
8	F01_ZSET_ST2_ST3A_ST4_ST6_007_Sample_20230815_102441.fsa

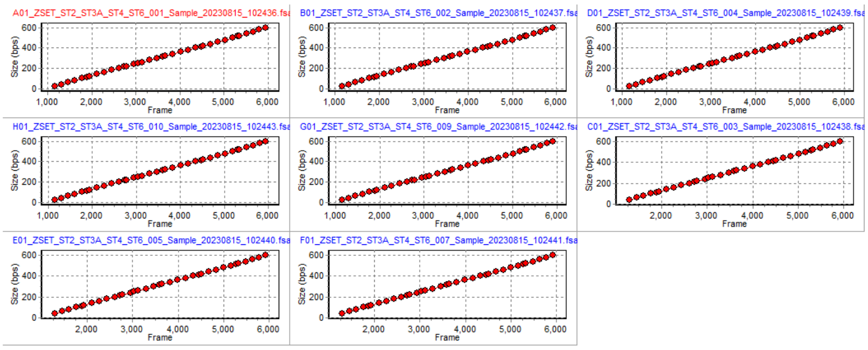

Supplementary Figure 3. Calibration chart of the sample during capillary electrophoresis.

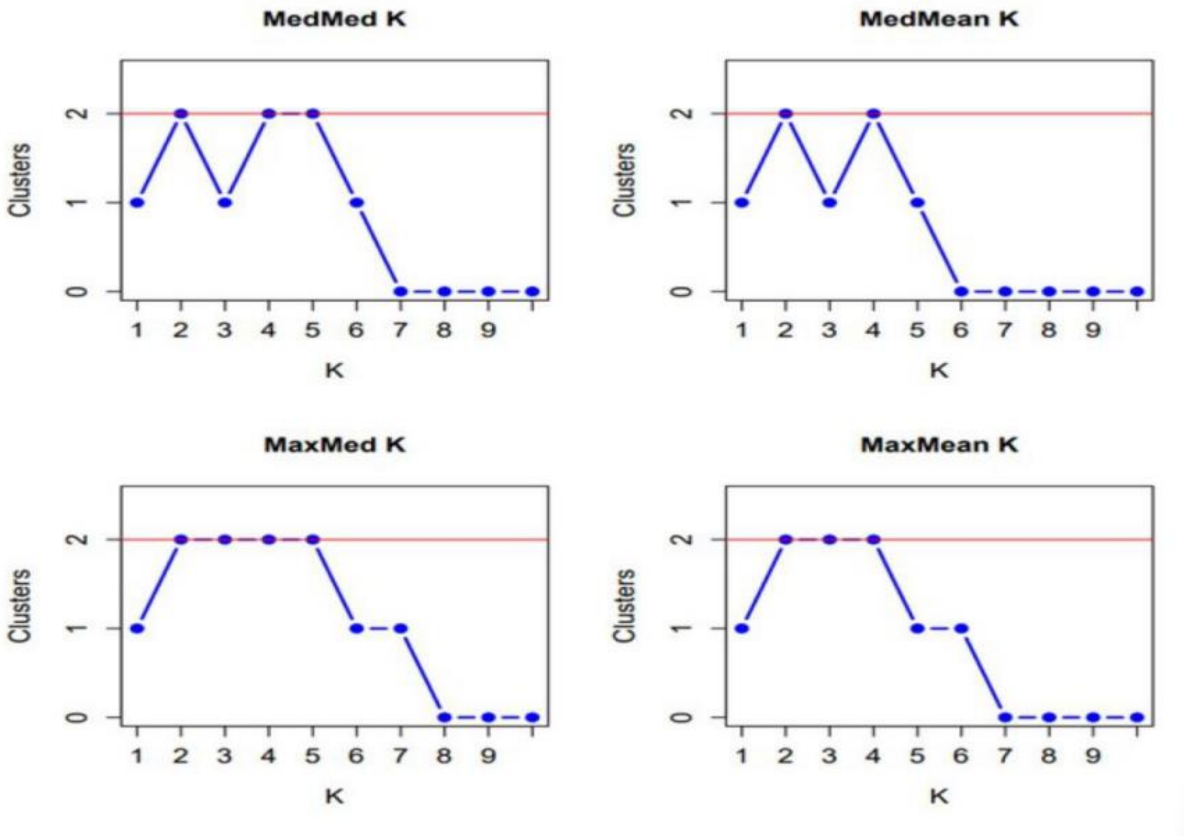

Supplementary Figure 4. Graphs displaying an optimum of two genetic clusters representing the six Zymoseptoria tritici populations based on the approach of (Puechmaille, 2016) where K = Population Cluster (1 - 9).
